# A community-led initiative to de-risk and advance Parkinson’s disease therapeutic targets

**DOI:** 10.1038/s41531-025-01039-3

**Published:** 2025-06-20

**Authors:** Alexandra Vaiana, Jonathan Behr, Ryan Birol, Cornelis Blauwendraat, Bradford Casey, Kushan Chowdhury, Martin Citron, Joshua Crapser, Victoria Dardov, Fiona Ducotterd, Sonya Dumanis, John Dunlop, Michelle Durborow, Brian Fiske, Jessica Golden, Jonas Hannestad, Wendy Hung, Jennifer Kemp, Robin Kleiman, Adam Knight, Andrew Koemeter-Cox, Bruce Leuchter, Bejamin A. Logsdon, Rita Marreiros, Julie E. Miller, Amanda Mitchell, Pooja Mukherjee, Grace Navarro, Matthew R. Nelson, Karoly Nikolich, Tom Otis, Nicole Polinski, Shima Rastegar-Pouyani, Alastair D. Reith, Ekemini Riley, Lee Rubin, Mina Ryten, Jessica Sadick, Tina Schwabe, Todd Sherer, Sarah Silvergleid, Andrew Singleton, Lara St. Clair, Jan Stoehr, David J. Stone, Julianna Sullivan, Nicole Tanenbaum, Elisa Tinelli, Kate Trimble, Yifei Wang, Stacie Weninger, Nicolás Wiggenhauser, Stephen Wood, Darryle Schoepp, Virginie Buggia-Prevot, Shalini Padmanabhan, Gaia Skibinski

**Affiliations:** 1 The Michael J. Fox Foundation for Parkinson’s Research, New York, NY USA; 2 SV Health Investors, Boston, MA USA; 3 Dementia Discovery Fund, Boston, MA USA; 4 Coalition for Aligning Science, Chevy Chase, MD USA; 5 Biotech Connection Bay Area, San Francisco, CA USA; 6 UCB Pharma, Brussels, Belgium; 7 Department of Neurology and Neurological Sciences, Stanford University School of Medicine, Stanford, CA USA; 8 Technome, Herndon, VA USA; 9 University College London, Great Britain, UK; 10 Aliada Therapeutics, Boston, MA USA; 11 Gain Therapeutics, Bethesda, MD USA; 12 University of California, San Franscisco, San Franscisco, CA USA; 13 Strategies for Open Science (Stratos), Santa Cruz, CA USA; 14 Myrobalan Therapeutics, Medford, MA USA; 15 Neuro.VC, San Franscisco, CA USA; 16 Neurvati Neurosciences, New York, NY USA; 17 Cajal Neuroscience Inc., Seattle, WA USA; 18 Chan Zuckerberg Biohub, San Franscisco, CA USA; 19 Departments of Neuroscience and Speech, Language and Hearing Sciences, University of Arizona, Tucson, AZ USA; 20 Independent Consultant, Seattle, WA USA; 21 University of California Berkeley, Department of Molecular and Cell Biology, Berkeley, CA USA; 22 Genscience LLC, New York, NY USA; 23 Deerfield Management, New York City, NY USA; 24 Department of Psychiatry, Stanford University School of Medicine, Stanford, CA USA; 25 Lario Therapeutics Ltd., Edinburgh, Scotland UK; 26 Memory and Aging Center, Weill Institute for Neurosciences, University of California San Francisco, San Francisco, CA USA; 27 Breckenfield Consulting Ltd., London, UK; 28 Department of Stem Cell and Regenerative Biology, Harvard University, Cambridge, MA USA; 29 UK Dementia Research Institute and Department of Clinical Neurosciences, School of Clinical Medicine, University of Cambridge, Cambridge, UK; 30 Department of Medical Genetics, University of Cambridge, Cambridge Institute for Medical Research, Cambridge, UK; 31 Valo Health Inc., Lexington, MA USA; 32 Nine Square Therapeutics, South San Franscisco, CA USA; 33 Schrödinger Inc., New York, NY USA; 34 Global Parkinson’s Genetics Program, Chevy Chase, MD USA; 35 AbbVie, Cambridge, MA USA; 36 Department of Microbiology and Immunology, Stanford University School of Medicine, Stanford, CA USA; 37 Golgi Neurosciences S.r.l, Milan, Italy; 38 Department of Nutritional Sciences and Toxicology, University of California, Berkeley, CA USA; 39 F-Prime Capital Partners, Boston, MA USA; 40 Interdepartmental Doctoral Program in Anthropological Sciences, Stony Brook University, Stony Brook, NY USA; 41 Neuron23 Inc., South San Franscisco, CA USA; 42 Independent Pharmaceutical Research Consultant, Ardmore, PA USA

**Keywords:** Drug discovery, Neuroscience

## Abstract

Identifying effective therapeutic targets for Parkinson’s disease (PD) is challenging, with no current disease-modifying therapies available. To address this, The Michael J. Fox Foundation for Parkinson’s Research launched the Targets to Therapies (T2T) initiative, uniting experts to prioritize and validate promising targets. T2T aims to develop validation strategies, create comprehensive target data profiles, and build tools to support drug development, ultimately accelerating the discovery of new therapies for PD patients.

## Introduction

Parkinson’s disease (PD) affects 6–11 million people worldwide, making it the second most common neurodegenerative disorder after Alzheimer’s disease^1^. To manage the hallmark motor symptoms of the disease, dopamine-directed therapies and surgical approaches (deep brain stimulation, focused ultrasound ablation) remain standard treatments^2–4^. Interventions addressing other motor complications and debilitating non-motor symptoms are available, but few are approved specifically for PD^3^. Despite extensive efforts, there are still no approved disease-modifying treatments that can effectively slow or halt the progression of PD.

The future remains promising for PD, as the therapeutic development pipeline is robust with approaches targeting the underlying biology with the intention to delay its progression. Indeed, recent estimates suggest more than half of treatments in current clinical testing seek disease modification^5–7^. Targets identified through genetic studies, such as alpha-synuclein, *LRRK2*, and *GBA1*, have been central to these efforts, and several programs are now testing agents directed against these targets in advanced phases of clinical testing^7^. Even if these initial approaches are successful in demonstrating disease slowing in some patients, given the disease’s diverse biology and heterogeneous nature, a broad range of therapeutics, alone or in combination, will likely be needed. As results from ongoing trials begin to emerge, it is clear that the overall pipeline remains risky and thus, it is crucial to continue exploring new therapeutic targets with the potential to modify disease progression and provide symptom relief. Expanding patient datasets combined with recent advances in genome- (GWAS), transcriptome- (TWAS) and proteome-wide association studies (PWAS), as well as with other methods, have resulted in many potential therapeutic targets^8–12^. However, many lack the necessary validation data to attract interest and investment in therapeutic development. Given the high costs for developing CNS-directed therapies, estimated at an average of $1 billion per drug, and the significantly longer development timelines for disease-modifying treatments compared to symptomatic ones, companies face substantial challenges^13,14^. These include the heightened risk of patent expiration stemming from prolonged clinical development and multiple trial failures. As a result, companies need to extensively de-risk and validate a target’s biological relevance and potential to impact disease progression before investing heavily.

Traditional validation approaches are critical, but they are often inefficient and fragmented, with limited coordination and replication leading to incomplete data packages around a target of interest. Moreover, if results are unpublished or kept proprietary, it can lead to costly and unnecessary duplication of research. This approach leaves many targets stranded and hinders their advancement beyond initial proof of concept and into therapeutic translation. The Michael J. Fox Foundation (MJFF) has a long history of working with the research and drug development communities to support validation of promising biological targets for PD^15–17^. To better address some of the challenges of traditional target validation and to prepare for the continued wave of newer biological insight coming from the research community, MJFF has launched a new program known as Target to Therapies (T2T). The T2T initiative adopts a community-connected approach including researchers, industry experts, and investors to prioritize and validate promising targets for PD. Key deliverables of T2T include the development of high-quality, validated target data packages to launch new industry programs or bolster existing ones. By doing so, T2T aims to accelerate the advancement of promising targets across the drug development pipeline and foster a new generation of diverse, robust PD therapeutics. Here we describe an overview of T2T and its first target prioritization effort to evaluate promising targets for target validation.

## The T2T engine for target prioritization and validation

**Fig. 1:**
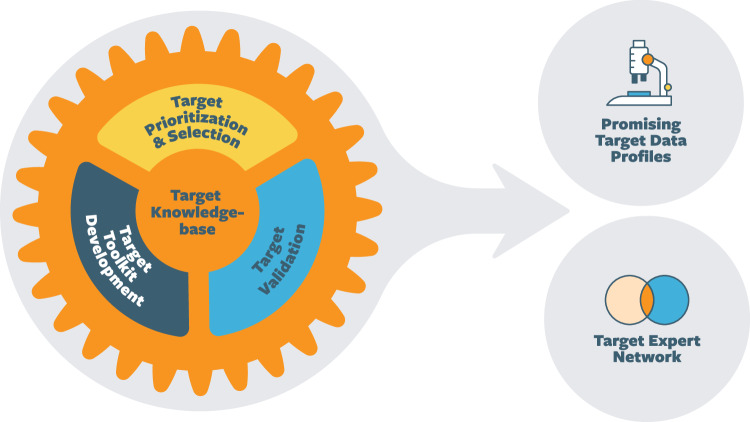
The engine driving T2T comprises three interconnected cores. Target Prioritization and Selection, Target Validation and Toolkit Development, and the Target Knowledge Base. The outcomes of this approach will be comprehensive target data packages that are well-positioned for therapeutic development and an informed community of researchers and investors with expertise on these targets, facilitating partnerships and potential investments to advance therapeutic development.

To accelerate targets across the translational pipeline, T2T will consist of three interconnected cores, forming a T2T “engine” designed as a sustainable and dynamic model to guide future target prioritization and validation efforts within MJFF (Fig. 1):

1. The Target Prioritization and Selection Core will create a scalable framework that leverages diverse data sources to prioritize promising PD targets for validation.
2. The Target Validation and Tool Kit Development Core will build target-specific toolkits and support validation efforts to build comprehensive target data profiles and tools that enhance confidence in drug development decision-making.
3. A Target Knowledge Base Core will develop a centralized platform to make resulting data and resources publicly accessible, generate target insights, and streamline data and resource sharing.

In early 2024, T2T established the Prioritization and Selection Core to develop a framework for evaluating and identifying priority targets for validation. Additionally, the Target Knowledge Base Core began developing a custom-built knowledge base to consolidate evaluated target data profiles, including those from the initial iteration of target evaluation and prioritization, with the goal of sharing them with the PD research community. With initial targets selected, T2T is now entering the first phase of Target Validation & Toolkit Development, to address data and resource gaps for the priority targets.

## Assembly of the target prioritization and selection core

Critical for the success of T2T was to first assemble a Prioritization and Selection Core team with diverse and proven expertise in PD drug development. We identified key stakeholders across academia, industry, and the investor community to guide the development of an objective framework to evaluate promising targets and conduct target diligence. The Core team consisted of 28 executives and scientific leaders from a wide range of biotech companies, large pharmaceutical companies, investment and venture capital firms, research institutes, nonprofits, and universities (Supplementary Fig. 1). Headed by three co-chairs with experience in large and small biotech, pharma, academia, and independent consulting, this Core team collaborated to establish a community-acceptable set of standards for evaluating and prioritizing targets while also capturing a broad view of potentially promising therapeutic targets.

## Target diligence and prioritization process

**Fig. 2:**
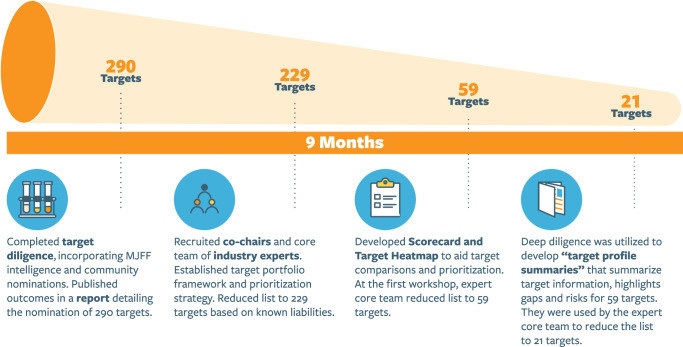
Milestones and timeline for prioritization and selection. Over a 9-month period, 290 targets were evaluated and prioritized through a staged approach involving multiple rounds of diligence. This process included the creation of target scorecards, heatmaps, and target profile summaries, which were used in successive rounds of evaluation to refine the list to 21 priority targets for initial de-risking validation efforts.

To build a sustainable and cooperative model for target selection and prioritization, we used a multi-stage approach. This began with broad community nominations of potential PD therapeutic targets, complemented by insights from MJFF's internal portfolio. Then the Prioritization and Selection Core performed two rounds of prioritization through a rigorous due diligence process supported by literature reviews and database searches on the targets (Fig. 2).

### Nomination of emerging targets for PD

**Fig. 3:**
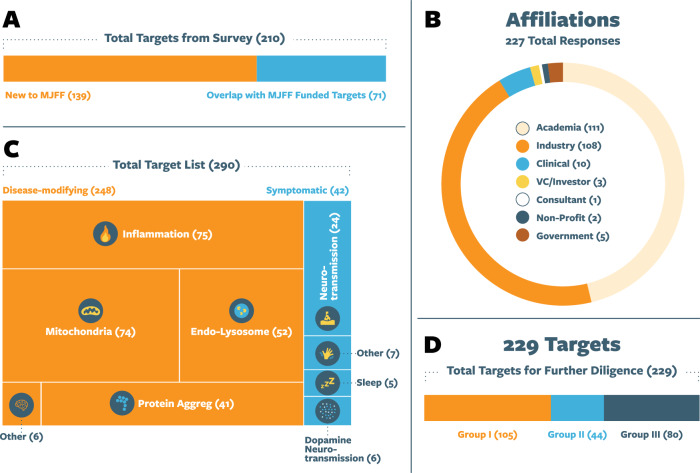
Summary of nominated targets and target list composition. **A** A total 210 targets were nominated, of which 71 overlapped with MJFF-funded targets, while 139 were new nominations. **B** Nominations were received from a diverse group of 227 stakeholders across various sectors. **C** The comprehensive list of 290 targets includes both disease-modifying and symptomatic targets. **D** Targets span different phases of the discovery pipeline, classified as: group I (no tool compounds available), group II (preclinical tool compounds available), and group III (drugs already in clinical studies for CNS or non-CNS indications).

We started by gathering broad community input through a survey to nominate targets for evaluation and received 227 responses from professionals across industry, academia, clinical practice, government, venture capital, consulting, and nonprofit organizations (Fig. 3A, B). The responses were combined and compared with a list of 151 targets from MJFF’s historical, funded research portfolio. Reflecting the value of our community-driven sourcing model, the survey identified 139 targets not yet seen in MJFF’s historical portfolio. These targets either had not previously been funded by MJFF or were nominated by singular groups or individuals, rendering them largely overlooked in previous prioritization efforts. Together, this resulted in a total of 290 targets which became the starting point for further due diligence and prioritization (Fig. 3C)^18^.

As a starting point for T2T, we chose to focus on targets nominated for their disease-modifying potential. We then further categorized these targets into broad biological pathways linked to PD pathology (Fig. 3C). As an initial quality check, we deprioritized targets without any data linking them to PD biology. This refinement resulted in a list of 229 targets for further diligence. To ensure the process remains current and evidence-driven, the prioritization and selection framework is intentionally dynamic, allowing targets to gain priority as new data emerges to enhance their therapeutic potential. Also, in subsequent iterations of prioritization and selection, we will expand to include targets aimed at symptomatic relief, in addition to those with disease-modifying potential.

### Categorizing targets by drug discovery progress

We next categorized each target based on the availability of target-specific tool compounds and if there were drugs already in development for the target. For tool compounds, we searched databases like MedChemExpress (MCE), Selleckchem, Google Patents, and PubMed, gathering data on drug names, therapeutic directionality, potency, off-target activities, and preclinical efficacy. Drugs in clinical trials were analyzed using Citeline Trialtrove. Based on this assessment, we categorized targets into three groups: group I (no tool compounds available), group II (pre-clinical tool compounds available), and group III (drugs already in clinical studies for CNS or non-CNS indications). The 229 targets included a balanced representation from each group and offered a range of targets, including those that could be quickly advanced if further validation data were promising and those needing more foundational supporting data (Fig. 3D).

### Assembling the light scorecard and first round of prioritization

**Fig. 4:**
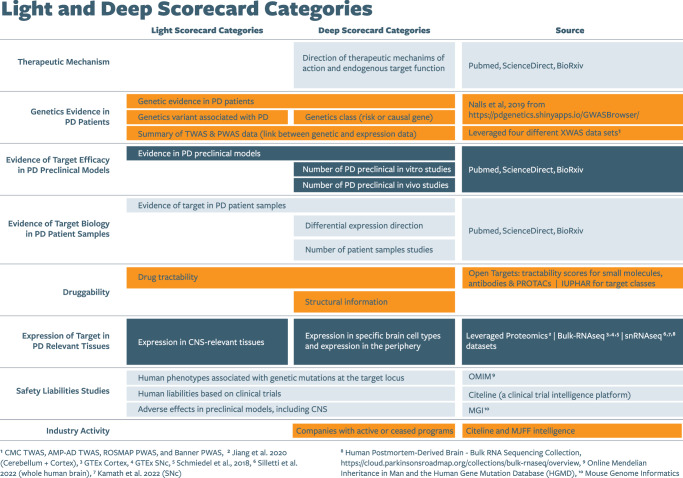
Scorecard categories enabled compiling of data diligence on the targets. The “light scorecard” categories were used in the initial round of prioritization and selection, while the “deep scorecard” expanded on these, providing additional diligence in each category for a more thorough evaluation of each target.

We used a two-stage prioritization process to narrow the list of 229 targets to those we would consider in our first round of validation efforts. This first stage of prioritization allowed us to rapidly assess each target’s role in the pathogenesis of PD and suitability for drug development. With input from the Target Prioritization and Selection Core, we first developed a “light scorecard” to systematically evaluate each target across six key evidence categories: genetic evidence in people with PD, efficacy in PD preclinical models, evidence of altered target biology in PD patient samples, target expression in CNS- and PD-relevant tissues, and target druggability and safety (Fig. 4).

Each target was evaluated for evidence of genetic risk variants in PD patients using genome-wide association study (GWAS) data from the Global Parkinson’s Genetics Program (GP2). Based on the strength of genetic association with PD, targets were classified into one of three categories: causal genes, risk genes, or genes with no known genetic association with the disease. We also used genome-wide, multi-omics association data from multiple sources (CommonMind Consortium TWAS, Accelerating Medicines Partnership-Alzheimer’s Disease TWAS, Religious Orders Study/Memory and Aging Project PWAS, and Banner PWAS) to evaluate the functional effects of the variants on the targets^19–21^. Additional literature searching and sourcing of data from bulk RNA-sequencing studies identified evidence for whether a target’s expression, activity, or localization was altered in PD patient samples, such as blood, brain tissue, or cerebrospinal fluid, or in CNS-relevant tissues impacted in PD^22–26^. Targets were considered expressed if their levels in the key tissue exceeded dataset-specific thresholds, as defined in each source. Expression dataset references are listed in Fig. 4. Evaluation of each target’s genetic and expression data relied on published analyses rather than conducting de novo analysis. We searched public literature databases (PubMed and BioRxiv) for existing target validation data from a range of PD preclinical models, including in vitro and in vivo models (Fig. 4). Using various sources that catalog therapies for specific target classes (e.g., Open Targets, IUPHAR), we determined druggability of each target by assessing if it belonged to established druggable classes (e.g., enzymes, G protein-coupled receptors, ion channels) and its suitability for existing therapeutic modalities (e.g., small molecules or antibody therapies). Targets likely requiring advanced technologies, such as gene editing or biologics, were not excluded based on druggability alone but were discussed further to address unique development considerations. Finally, target liability risk was evaluated using three complementary approaches. First, human liability was assessed by examining phenotypes associated with genetic mutations at the target locus, using data from Online Mendelian Inheritance in Man (OMIM). Second, preclinical safety signals, including adverse effects in the CNS, were reviewed using data from the Mouse Genome Informatics (MGI) database. Lastly, clinical safety was evaluated by analyzing records in Citeline TrialTrove to identify any discontinued or terminated trials potentially linked to safety concerns involving the target. Insights across these six categories for each target were reviewed at an in-person workshop leading to a prioritized list of 59 targets (Supplementary Tables 1–4).

### Enhancing a deep scorecard, generating target profile summaries, and final prioritization

With continued feedback from Target Prioritization and Selection Core members, we initiated the second stage of prioritization by performing a more comprehensive diligence process of the 59 targets. This included a deeper review of select light scorecard categories and several additional criteria (Fig. 4), and we also identified gaps and risks to de-risk the target for therapeutic development. For example, for targets with evidence of biology in PD patient samples, we confirmed the type of sample, the assays employed, and the observed alterations in target biology. A secondary analysis of PD preclinical model efficacy confirmed whether studies used PD-specific models with direct target modulation (e.g., genetic, biological, or pharmacological methods) and the experimental assays, endpoints, and outcomes of target modulation in these models. The endogenous expression of the target was broadened to include a wide range of CNS and peripheral cell types, utilizing single-cell and bulk RNAseq datasets (Fig. 4)^27^.

Deeper diligence also sought to define a target’s therapeutic mechanism of action, emphasizing therapeutic directionality, whether increasing or decreasing target activity or expression is linked to PD and thus how a therapy might best intervene. Therapeutic directionality was evaluated based on several data sources, including genetic evidence, PD patient-derived sample information, gene expression studies (such as PWAS and TWAS), and the broader body of evidence from target efficacy studies in preclinical PD models. Published data on endogenous target functions, availability of structural information (e.g., crystal structures), and target expression in relevant peripheral tissues and specific brain cell types further rounded out our assessment. Finally, we searched for evidence of active or historical industry engagement with each target, including whether a therapeutic asset was CNS or non-CNS penetrant (Fig. 4). While there was broad consensus on the categories to be included in both scorecards, expert opinions varied significantly on the relative importance each category should hold. This divergence highlighted the inherent complexity of establishing evaluation criteria and reinforced the need for a flexible framework that can accommodate a range of expert perspectives.

**Fig. 5:**
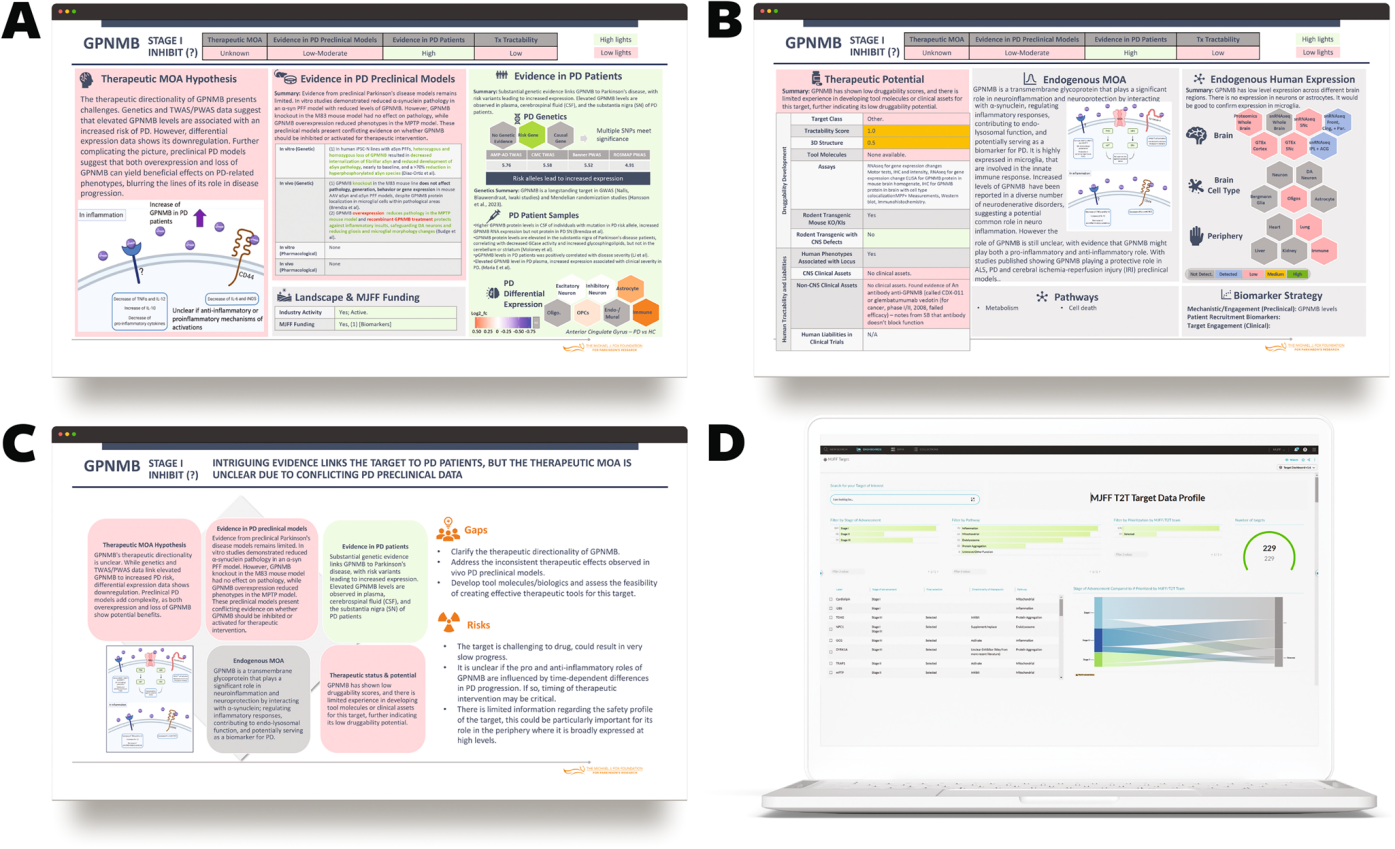
Target profile summaries and knowledge base “illuminate”. **A**–**C** These represent one of the 59 target profile summaries created for the prioritized targets. **A**, **B** The first two slides summarize target diligence across scorecard categories such as therapeutic mechanism of action (MOA), efficacy evidence in preclinical PD models, patient biology, and therapeutic potential. **C** The third slide provides a summary of each key diligence area, along with a list of gaps in the target profile and associated risks in therapeutic development for the target. **D** A screenshot of the target profile page in the knowledge base displays the data collected during this round of prioritization and selection.

We used this deeper diligence to update information for each target and to create detailed target profile summaries for all 59 targets. These profile summaries were designed to mirror the target selection process commonly used by industry when evaluating target opportunities, including data for each scorecard category and a summary of each key diligence area, data gaps in the target profile, and potential risks in therapeutic development for the target (Fig. 5A–C).

During a two-day workshop, Core team members used the deep scorecards and detailed target profile summaries to systematically refine the list and identify 21 prioritized targets (21 prioritized targets in bold text in Supplementary Tables 1–4). The selection process was guided by the goal of building a balanced and strategically diverse portfolio, one that spans a range of biological pathways implicated in PD as well as varying levels of target maturity across the drug development pipeline. This approach ensured that the final set of prioritized targets included both well-characterized targets with translational potential and emerging targets that may benefit from additional foundational research. For each target, we identified additional gaps in the target profile including the availability of target-directed compounds and mapped out preliminary de-risking validation plans. We also discussed additional insights into industry activity and sought recommendations for key experts for each target.

## Improving the prioritization and selection core for future evaluations

Through T2T, we developed a framework to evaluate and prioritize targets for validation, leveraging a broad, community-informed model that integrates expertise from academia, industry, investors. Our goal is to build on the framework to repeat and refine prioritization and selection of additional targets in a scalable and sustainable manner. In the next round of prioritization, we will incorporate progression-related genetic data to provide a more comprehensive genetic understanding of target relevance in PD^28,29^. We will also integrate biomarker data into our strategy, recognizing that early consideration of biomarkers is crucial to the success of future clinical trials. We plan to align T2T efforts with ongoing MJFF and other biomarker initiatives to further inform the target prioritization and validation process. These efforts will focus on identifying biomarkers for target engagement, pharmacodynamics, patient stratification, and efficacy in both current and future disease-modifying trials. In parallel, each prioritized target will undergo assessment for its biomarker development strategy during validation de-risking efforts, including identification of any enabling tools that could accelerate this process and support future clinical decision-making.

This initial selection process exclusively prioritized targets with disease-modifying potential. Subsequent efforts will implement a focused strategy for identifying targets that could effectively alleviate the range of symptoms that occur with PD and its progression. This dedicated track of T2T will aim to uncover therapeutic targets to modify or prevent associated symptoms such as cognitive decline, gait and balance impairments, sleep disturbances, or anxiety, among others, that may require distinct therapeutic strategies for improvement.

**Fig. 6:**
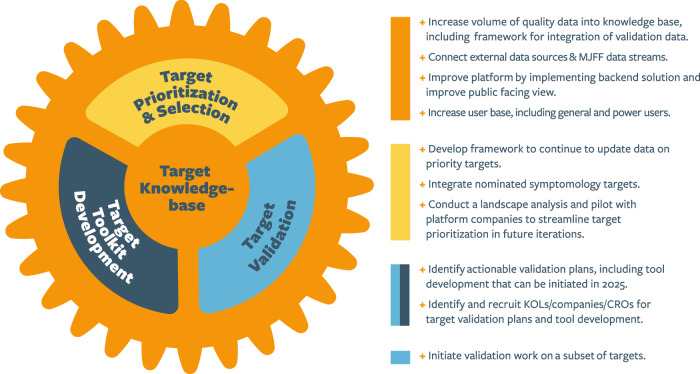
Next steps for T2T. An outline of the immediate actions for T2T, including target prioritization and selection, target validation, and the development of the knowledge base.

As a dynamic initiative, T2T will evaluate new and re-evaluate existing target packages based on emerging data, ensuring the most promising targets enter the T2T validation engine (Fig. 6). While this process may introduce certain biases, we have taken decisive steps to minimize them by establishing a robust framework for target selection. This framework, developed through a community-led initiative, can be effectively adjusted based on the guidance and insights from the PD research and drug development community. In future iterations, we will leverage emerging technologies, including AI, to streamline data integration, enhance target evaluation, and share insights with the PD community. Scaling and automation will continue to prioritize high-quality data, expert curation, and therapeutic impact over volume.

## Advancing to the next phase of the T2T initiative

With its initial list of 21 priority targets, T2T will focus on initiating validation and toolkit development. A dedicated Validation Core, comprising experts in PD biology, target validation, and CNS drug development, is being assembled to collaboratively develop robust data validation roadmaps, define milestone-based inflection points, and implement clear and effective data sharing protocols for tools and resources. In parallel, a Target Toolkit Development Core, including specialists in tool, assay, and model development, will generate preclinical tools for each target, overcoming potential roadblocks and enabling accelerated progress (Fig. 6).

Critical to the scalability and efficiency of T2T, the validation and toolkit plan will focus on shared validation approaches, leveraging common tools or models for targets with similar gaps or within the same biological pathway (e.g., endolysosomal targets). MJFF will launch funding programs to support validation work on the priority targets and development of key tools, assays and models needed to initiate and support validation and drug discovery efforts. To ensure alignment with T2T goals and uphold data quality, validation efforts will be regularly assessed by key stakeholders and independent experts through team meetings and progress reports. These evaluations will focus on scientific progress, rigor, data reproducibility and evolving therapeutic potential of each target.

To streamline data and resource sharing, including the target data profiles, a centralized knowledge base platform is under development (Fig. 5D). An initial version has been shared with T2T core members and stakeholders to collect feedback critical for enhancing its functionality. In the future, this knowledge base will be made accessible to the broader PD community. It will offer comprehensive profiles for all evaluated targets, providing transparency into their status and their supporting evidence. As new data becomes available or additional targets are nominated, they will be integrated into the platform.

T2T’s broader success will be measured by the initiation or acceleration of PD drug discovery programs linked to its prioritized targets. These programs will have a greater chance of therapeutic success based on robust, well-validated data linking a target to PD-relevant biology. Over the course of the 2–3-year program, the T2T initiative will uphold The MJFF core principles of open science and data transparency. Program updates and progress on target profiles will be made publicly available on the T2T main website (Targets to Therapies Initiative | Parkinson’s Disease]), the T2T knowledge base, and open access platforms enabling the broader research community to stay informed and contribute feedback to the initiative. Moreover, while T2T focuses exclusively on PD, the data from this effort, along with our target evaluation frameworks, can be leveraged to inform research and therapeutic development in other CNS indications, creating opportunities for broader impact in the field of neurodegenerative research.

As MJFF continues to lead efforts in advancing the discovery of new therapies for PD, the T2T initiative remains a dynamic and scalable component of the Foundation’s portfolio, focused on de-risking emerging therapeutic targets. By systematically evaluating the biological relevance, translatability, and therapeutic potential of these targets, T2T aims to build the confidence needed to move them into drug development and, ultimately, human testing, where their clinical impact can be fully assessed. This initiative is one of several MJFF programs aimed at accelerating therapeutic innovation in PD, and the Foundation will continue to foster collaborations with other programs to advance this goal. We encourage the research community to visit our website for updates on T2T and other emerging opportunities as new programs are launched.

## Supplementary information

Supplementary Information

## Data Availability

No datasets were generated or analyzed during the current study.
