# Supplementary material for: A community-led initiative to de-risk and advance Parkinson’s disease therapeutic targets
Source: NPJ Parkinsons Dis. 2025 Jun 20;11:179. doi: 10.1038/s41531-025-01039-3 (PMC12181325; doi:10.1038/s41531-025-01039-3)
Supplement: Supplementary file 1 — Supplementary Information [file 41531_2025_1039_MOESM1_ESM.pdf]

Supplementary Figure 1. Breakdown of P&S Core Team Members by Affiliation. *The Core is composed of 36 members including the P&S co-chairs, industry scientists, investors, subject matter experts, and BCBA consultant scientists.*

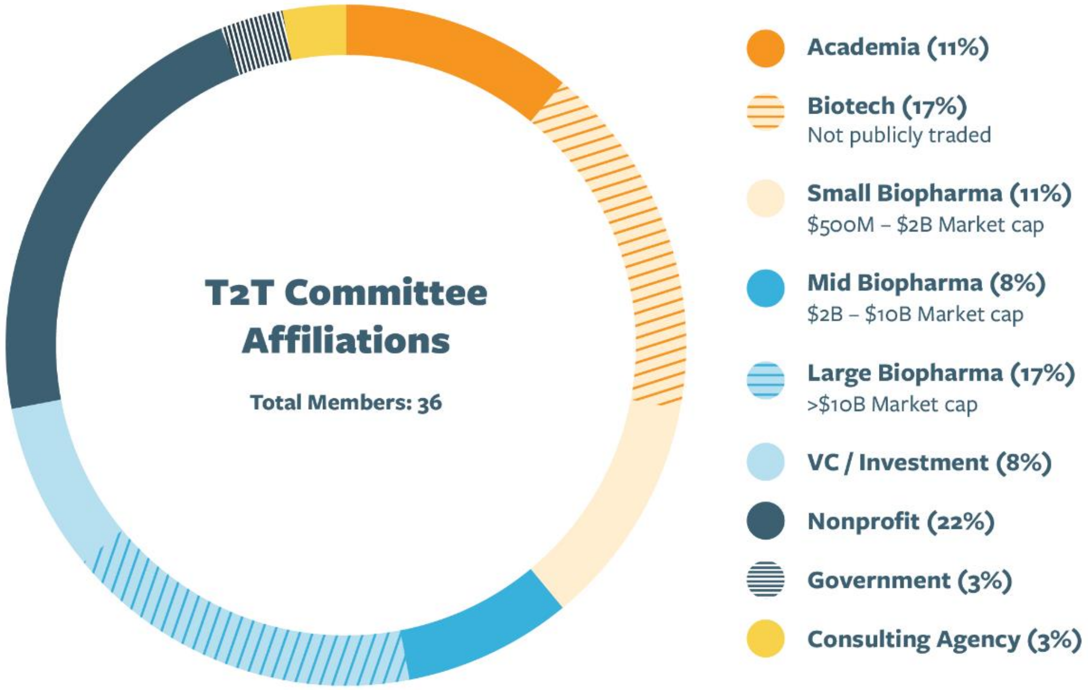

Supplementary Table 1. Endolysosomal targets identified among the 59 prioritized by Core Team members following the first workshop. *Targets are listed alphabetically. Bolded names indicate endolysosomal targets that were included among the 21 top-priority selections for the first round of validation studies.*

59 Target list: Endolysosome

Target	Protein Name	UniProt IDs	Ensembl ID	Group of Advancement
**ATP13A2**	**Polyamine-transporting ATPase 13A2; ATPase Cation Trasporting 13A2**	**Q9NQ11**	**ENSG00000159363**	**Group I**
CSNK2B	Casein kinase 2 beta	P67870	ENSG00000204435	Group III
**CTSB**	**Cathespin B**	**P07858**	**ENSG00000164733**	**Group III**
GALC	Galactosylceramidase	P54803	ENSG00000054983	Group II
**GBAP1**	**glucosylceramidase beta pseudogene 1**	**not available**	**ENSG00000160766**	**Group I**
**GPNMB**	**Transmembrane glycoprotein NMB**	**Q14956**	**ENSG00000136235**	**Group I**
GRN	Progranulin/Granulin Precursor/PGRN	P28799	ENSG00000030582	Group III
**MCOLN1**	**mucolipin TRP cation channel 1; TRPML1**	**Q9GZU1**	**ENSG00000090674**	**Group II**
NPC1	NPC Intracellular Cholesterol Transporter 1	O15118	ENSG00000141458	Group I
PSAP	Prosaposin	P07602	ENSG00000197746	Group I
RAB32	RAB32, Member RAS Oncogene Family	Q13637	ENSG00000118508	Group I
RILPL1	Rab interacting lysosomal protein like 1	Q5EBL4	ENSG00000188026	Group I
RIT2	Ras Like Without CAAX 2	Q99578	ENSG00000152214	Group I
SCARB2	LIMP2	Q14108	ENSG00000138760	Group I
SMPD1	Sphingomyelin Phosphodiesterase 1	P17405	ENSG00000166311	Group II
SPTLC2	Serine Palmitoyltransferase Long Chain Base Subunit 2	O15270	ENSG00000100596	Group I
SYNJ1	synaptojanin-1	O43426	ENSG00000159082	Group I
TFEB	Transcription Factor EB	P19484	ENSG00000112561	Group II
**TMEM175**	**transmembrane protein 175**	**Q9BSA9**	**ENSG00000127419**	**Group II**
TMEM230	Transmembrane Protein 230	Q96A57	ENSG00000089063	Group I
VPS13C	vacuolar protein sorting 13 homolog C	Q709C8	ENSG00000129003	Group I
**VPS35**	**VPS35 retromer complex component**	**Q96QK1**	**ENSG00000069329**	**Group I**

Supplementary Table 2. Mitochondrial targets identified among the 59 prioritized by Core Team members following the first workshop. *Targets are listed alphabetically. Bolded names indicate mitochondrial targets that were included among the 21 top-priority selections for the first round of validation studies.*

59 Target list: Mitochondrial

Target	Protein Name	UniProt IDs	Ensembl ID	Group of Advancement
**BECN1**	**Beclin1**	**Q14457**	**ENSG00000126581**	**Group I**
**CACNA1D**	**Voltage dependent L-type calcium channel subunit alpha-1D (CaV1.3)**	**Q01668**	**ENSG00000157388**	**Group I**
DYRK1A	Dual Specificity Tyrosine Phosphorylation Regulated Kinase 1A	Q13627	ENSG00000157540	Group III
FBXO7	F-Box Protein 7	Q9Y3I1	ENSG00000100225	Group I
GPR37	Prosaposin receptor GPR37; G Protein-Coupled Receptor 37	O15354	ENSG00000170775	Group I
HMOX1	Heme oxygenase 1	P09601	ENSG00000100292	Group III
KANSL1	KAT8 regulatory NSL complex subunit 1	Q7Z3B3	ENSG00000120071	Group I
mPTP	Mitochondrial permeability transition pore	not available; complex	not available	Group II
PARK7	Parkinson disease protein 7/DJ1	Q99497	ENSG00000116288	Group II
TRAP1	TNF Receptor Associated Protein 1	Q12931	ENSG00000126602	Group II
USP15	ubiquitin specific peptidase 15	Q9Y4E8	ENSG00000135655	Group I
**USP30**	**ubiquitin specific peptidase 30**	**Q70CQ3**	**ENSG00000135093**	**Group III**
ZNF746	zinc finger protein 746; PARIS	Q6NUN9	ENSG00000181220	Group I

Supplementary Table 3. Protein aggregation targets identified among the 59 prioritized by Core Team members following the first workshop. *Targets are listed alphabetically. Bolded names indicate protein aggregation targets that were included among the 21 top-priority selections for the first round of validation studies.*

59 Target list: Protein Aggregation

Target	Protein Name	UniProt IDs	Ensembl ID	Group of Advancement
AIMP2	Aminoacyl TRNA Synthetase Complex Interacting Multifunctional Protein 2	Q13155	ENSG00000106305	Group II
DNAJC13	DnaJ Heat Shock Protein Family (Hsp40) Member C13/Rme8	O75165	ENSG00000138246	Group I
EIF2AK1	Eukaryotic Translation Initiation Factor 2 Alpha Kinase 1	Q9BQI3	ENSG00000086232	Group I
KLK6	Kallikrein Related Peptidase 6	Q92876	ENSG00000167755	Group I
**MAPT**	**microtubule associated protein tau**	**P10636**	**ENSG00000186868**	**Group III**
**OGA**	**O-GlcNAcase; MGEA5**	**O60502**	**ENSG00000198408**	**Group III**
**TGM2**	**Transglutaminase 2 (TG2)**	**P21980**	**ENSG00000198959**	**Group III**

Supplementary Table 4. Inflammation targets identified among the 59 prioritized by Core Team members following the first workshop. *Targets are listed alphabetically. Bolded names indicate inflammation targets that were included among the 21 top-priority selections for the first round of validation studies.*

59 Target list: Inflammation

Target	Protein Name	UniProt IDs	Ensembl ID	Group of Advancement
CD38	CD38	P28907	ENSG00000004468	Group III
CD84	CD84	Q9UIB8	ENSG00000066294	Group I
**CDK5**	**Cyclin Dependent Kinase 5**	**Q00535**	**ENSG00000164885**	**Group II**
DNAJC6	DnaJ Heat Shock Protein Family (Hsp40) Member C6/Park19; Auxilin	O75061	ENSG00000116675	Group I
FYN	FYN Proto-Oncogene, Src Family Tyrosine Kinase	P06241	ENSG00000010810	Group III
**GCG**	**GLP-1 (Glucagon-like peptide-1)**	**P01275**	**ENSG00000115263**	**Group III**
HLA-DRB5	Major Histocompatibility Complex, Class II, DR Beta 5	Q30154	ENSG00000198502	Group I
**NFE2L2**	**NFE2 Like BZIP Transcription Factor 2; NRF2**	**Q16236**	**ENSG00000116044**	**Group III**
NLRP12	NLR Family Pyrin Domain Containing 12	P59046	ENSG00000142405	Group I
NR4A2	nuclear receptor subfamily 4 group A member 2; Nurr1	P43354	ENSG00000153234	Group II
**NOD2**	**Nucleotide Binding Oligomerization Domain Containing 2**	**Q9HC29**	**ENSG00000167207**	**Group I**
PAM	Peptidylglycine Alpha-Amidating Monooxygenase	P19021	ENSG00000145730	Group I
**PARP1**	**Poly[ADP-ribose] polymerase 1**	**P09874**	**ENSG00000143799**	**Group III**
SOD1	superoxide dismutase 1	P00441	ENSG00000142168	Group III
**STING1**	**stimulator of interferon response cGAMP interactor 1; TMEM173**	**Q86WV6**	**ENSG00000184584**	**Group II**
**TLR2**	**toll like receptor 2**	**O60603**	**ENSG00000137462**	**Group III**
**TREM2**	**Triggering Receptor Expressed On Myeloid Cells 2**	**Q9NZC2**	**ENSG00000095970**	**Group III**
